# Consensus or Deadlock? Consequences of Simple Behavioral Rules for Coordination in Group Decisions

**DOI:** 10.1371/journal.pone.0162768

**Published:** 2016-09-28

**Authors:** Helen F. McCreery, Nikolaus Correll, Michael D. Breed, Samuel Flaxman

**Affiliations:** 1 Department of Ecology and Evolutionary Biology, University of Colorado, Boulder, Colorado, United States of America; 2 Department of Computer Science, University of Colorado, Boulder, Colorado, United States of America; Arizona State University, UNITED STATES

## Abstract

Coordinated collective behaviors often emerge from simple rules governing the interactions of individuals in groups. We model mechanisms of coordination among ants during cooperative transport, a challenging task that requires a consensus on travel direction. Our goal is to determine whether groups following simple behavioral rules can reach a consensus using minimal information. Using deterministic and stochastic models, we investigate behavioral factors that affect coordination. We define and investigate three types of behavioral rules governing individual behavior that differ in the information available: individuals either 1) have no information, 2) can measure transport success, or 3) measure success while also knowing whether they are aligned with the majority. We find that groups break deadlocks only if individuals more readily give up when they are going against the majority, corresponding to rule type 3 –such groups are “informed.” These behavioral rules succeed through positive and negative feedbacks that are implemented in our model via a single mechanism: individuals only need to measure the relative group sizes to make effective decisions. We also find that groups reach consensus more quickly if they have either a shared bias, high sensitivity to group behavior, or finely tuned persistence. Each of these is a potential adaptation for efficient cooperative transport. This flexibility makes the behavioral rules in the informed case relatively robust to deficiencies in the individuals’ capabilities. While inspired by ants, our results are generalizable to other collective decisions with deadlocks, and demonstrate that groups of behaviorally simple individuals with no memory and extremely limited information can break symmetry and reach a consensus in a decision between two equal options.

## Introduction

Across organizational scales, the patterns and complexity of many biological systems emerge from groups of individuals obeying relatively simple rules, often without a leader [1]. Rules typically apply to individuals interacting with their neighbors, and exploit positive and/or negative feedback mechanisms leading to coordinated group dynamics [1]. Rules do not have to be simple, but if robust, efficient coordination is possible with simple rules, there is no need for complex individual behaviors to evolve. Interest in discovering rules for collective behavior has produced a rich literature, and there has been particular interest in group decision making [2–5]. This includes nest-site selection decisions in honeybees and *Temnothorax* ants [6–9], decisions by groups of neurons in brains [10], decisions in non-neuronal organisms [11], and more. Ant colonies are particularly well suited to studies of collective behavior because workers can be easily observed and manipulated, and indeed, pheromone trail formation in ants is a classic study system for self-organized decision making (e.g. [1,12]).

In collective decisions, groups that deadlock–having approximately equal numbers of individuals aligned with each choice–fail to form consensus. This can result in a split decision, or no decision at all. For some types of decisions this can be catastrophic, and there are behavioral mechanisms to prevent deadlocks in these cases. For example, split decisions during nest-site selection in honey bees can result in colony death [7,13], and the “stop signal” has evolved to prevent such splits [9,14,15]. This stop signal is a negative feedback mechanism and, along with a positive feedback mechanism (advertising), ensures that colonies can break deadlocks to choose a single nest site [14]. In fact, colonies can choose a single site even when the options are of equal quality; this is an example of symmetry breaking. A substantial body of research has focused on symmetry breaking in various taxa including honey bees, ants, cockroaches, and more (e.g. [9,16–18], reviewed in [19]). Symmetry among choices makes deadlocks more likely, and the ability to overcome deadlocks is a crucial component of any collective decision in which a group must choose a single option.

A collective decision that is particularly prone to deadlocks occurs during cooperative transport in ants. Cooperative transport is the movement of large objects such as food items, intact, by multiple individuals [20], and it requires making one or more decisions about travel direction. Workers of some ant species collaborate to carry objects many thousands of times their mass [21–25]. This requires a high degree of coordination across many individuals, and ant species vary substantially in their ability to coordinate. Some species move objects rapidly toward their nests, while others are categorized as uncoordinated, having many deadlocks, with workers pulling in opposing directions for minutes or hours [25,26]. Even in species with efficient cooperative transport, short-lived deadlocks occur [24,25]. Deadlocks may happen if individuals have conflicting information about the direction of the nest, or if the group encounters an obstacle blocking the nest direction, requiring a new decision. Deadlocks may be more likely in cooperative transport than other decisions because cooperative transport groups are often relatively small. Larger groups are less affected by the behavior of single individuals. Split decisions are impossible in cooperative transport because group members are physically tethered together by the object they are attempting to carry, so deadlocked groups are stuck. Thus, deadlocks in cooperative transport are also conspicuous. The fact that deadlocks are common and conspicuous makes cooperative transport an ideal task for studying the resolution of deadlocks in collective decisions.

Prior research has revealed aspects of cooperative transport, including selection pressures, ecology, recruitment, and more (reviewed in [20,24,25]). This previous research has also included detailed descriptions and models of cooperative transport, and in some cases models have been compared with empirical data [24,27–29]. But these studies have not focused on comparing alternative behavioral rules for overcoming the coordination challenge; thus, our understanding of behavioral rules for deadlock breaking, and for cooperative transport generally, is limited. Some investigators have suggested that ants in groups use the same rules as individual transporters (reviewed in [24]). However, if rules for individual transport were sufficient, one would expect most ant species to be efficient at cooperative transport. This is not the case [25,30], and it is reasonable to think that efficient cooperative transporters have behavioral rules tuned to this task. What behavioral mechanisms separate the coordinated from the uncoordinated transporters?

We use a proof-of-concept model [31] to investigate the behavioral rules, information, and minimum complexity of individuals required in order to break deadlocks. Deadlock breaking has previously been studied in decisions with positive and negative feedback mechanisms (e.g. [14]). Here, we set out to determine if simple individuals employing just one feedback or even no feedbacks can break deadlocks. We model three broad categories of behavioral rules in both deterministic and stochastic contexts. These sets of rules differ in the kinds of information we allow individuals to perceive and the ways this information is used by individuals. We model one spatial dimension, so we examine a decision between two options for direction of travel: left or right. Our goal is not to identify the exact rules employed by all ants, but rather to explore the simplest behaviors and minimum information required to break deadlocks. Thus, we leave the comparison of our predictions with empirical patterns of transport for future research. Like other proof-of-concept models, the value in this work is that it tests the logic of verbal hypotheses and creates predictions that can be empirically tested [31]. Our investigations generate hypotheses for cooperative transport adaptations and offer insights into consensus decisions in other groups. The broad modeling approach we employ has been used extensively to elucidate behavior that is difficult to measure in collective systems, including social insects, robots, and beyond (e.g. [14,32,33,19,34,35]).

We use this approach to answer two primary questions. First, can realistic, simple behavioral rules reliably overcome deadlocks? As part of this question, we look at what information individuals must minimally receive. Second, what effects do persistence (maximum engagement time with the object) and sensitivity to information have on coordination? In answering these questions we generate hypotheses for cooperative transport adaptations and provide insight into the factors that affect deadlocks during cooperative transport, and during other collective decisions.

## Models

### Assumptions

We are interested in the minimum information and complexity requirements for deadlock breaking. We therefore assume individuals have minimal capabilities. As described below, we allow them little information. Our simulated ants also have no memory, in that they do not use information from past experiences to shape future behaviors. Real ants have more capabilities and information than the simulated ants in our models, but to find minimum requirements, we exclude several sources of information that have been demonstrated in one or more ant species. We further simplify real cooperative transport efforts by assuming that all ants are identical.

Ants sense a wide range of stimuli (e.g. [36–38]), though workers of a single species likely can sense only a subset of the total possible information. There are several ways that workers in a cooperative transport group might gain information about what others in the group are doing. They could communicate with one another, but while workers recruit additional help to the object to be carried, often with pheromone trails [20], there is no evidence of direct communication among ants after the recruitment phase. A simpler possibility is that workers communicate indirectly through the object being carried, an example of stigmergy. This stigmergy mechanism has been hypothesized for ant groups by Kube and Bonabeau (2000) and others [20,24,39]. This indirect communication does not require an evolved signal, as workers simply detect physical cues that necessarily arise when forces are applied to an object. In terms of the kinds of information available in our model, we only consider a narrow set of information that could plausibly be transmitted through the object itself. Specifically, the maximum information we allow individuals to receive about group members’ behavior is the *relative sizes* of the groups aligned left and right. This information could be transmitted through the object via the magnitude and direction of the object’s movements and/or deformations [20,39], or there are other possible mechanisms for individuals to estimate relative group sizes, such as direct communication. Our model thus serves as a logical test of the hypothesis that this minimal information is sufficient to break deadlocks. Our assumptions are appropriate based on existing literature regarding complexity requirements for group decisions and hypotheses specific to cooperative transport [11,20].

### Deterministic Model

**Fig 1:**
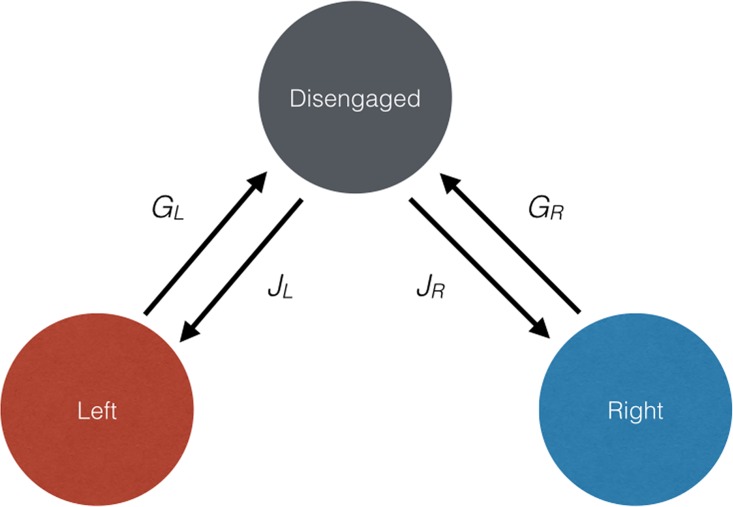
Model diagram. Individuals belong to one of three behavioral states: moving left, moving right, or disengaged. Individuals move between these states at rate constants *G*_*L*_, *G*_*R*_, *J*_*L*_, and *J*_*R*_.

We developed a deterministic, ordinary differential equations (ODE) model that simulates the average behavior of individuals. The model is Markovian–individuals have no memory–but non-linear. We model movement in one spatial dimension implicitly and we use continuous time and continuous abundances of individuals (but see individual-based model below). Individuals are identical, and the total number is fixed at 20; for some analyses we explored the effect of changing group size analytically and by evaluating groups with a total of 6 or 200 individuals. Having a fixed number of individuals is appropriate because the number of workers that can participate in cooperative transport will be limited by the number of grasping points on the object. Furthermore, the behavioral states we model allow varying numbers of individuals to be engaged with the object at any one time. Specifically, our model assumes that each individual occupies one of three mutually exclusive behavioral states: 1) trying to move the object to the left, 2) trying to move the object to the right, or 3) disengaged from the object (Fig 1). We do not distinguish between pushing and pulling; individuals pushing from the left and pulling from the right are both in the “move right” behavioral state. Individuals move from the disengaged state to an active state by “joining,” and from one of the active states to the disengaged state by “giving up.” Individuals do not move directly between the two active states, however, individuals can immediately re-join after giving up. The transition rates are important model parameters that govern the number of individuals in each behavioral state over time. We examine these abundances to see the extent to which the group converges on a single direction under the parameters of a specific model run.

#### Joining

Disengaged individuals join the transport efforts to the left and right with rate constants *J*_*L*_ and *J*_*R*_, respectively. The realized joining rates depend on the number of disengaged individuals: the instantaneous joining rate for the left state is *J*_*L*_ multiplied by the number of disengaged ants. We assume the joining rate constants do not change in time but may differ from each other, i.e. individuals may join the “move left” behavioral state at a higher rate than the “move right” state. If *J*_*L*_ and *J*_*R*_ are not equal, this ensures a directional bias, which is how we represent individuals having information about the direction of the goal.

In real ants, directional cues about the location of the nest come from one or more sources, such as pheromone trails, visual navigation, or path integration [5, 6]. Whatever the sensory modality may be, we assume this information is not perfect. That is, even if there is a directional bias, some individuals still choose the other direction (i.e., *J*_*L*_, *J*_*R*_ > 0). Joining rate constants do not vary during the transport effort; for example, we assume groups are not capable of altering their bias in favor of the “winning” direction (here we use the “winning” direction to indicate simply the direction that has more individuals). This makes sense given our conservative assumptions about individuals’ memory and sensory capabilities: individuals that are disengaged, and therefore not in contact with the object to sense information transmitted through it, cannot perceive which direction is winning and have no memory about which direction was winning when they were last engaged.

#### Giving-up

Individuals in the active behavioral states (left and right) give up at rate constants *G*_*L*_ and *G*_*R*_, respectively. We model three sets of behavioral rules for giving up rates. These sets of rules also differ in the kinds of information individuals act upon (Table 1). We do not suggest that all of the variation in cooperative transport behavior in ants is captured by these three sets of rules; rather, we explore these rules to see if such simple rules are sufficient to break deadlocks.

**Table 1:** Modeled sets of behavioral rules and information required for each.

Rule	Description	Information used
**“Uninformed”**	If in one of the active states, give up (become disengaged) at a constant rate	None
**“Oblivious”**	If in one of the active states, give up more readily when transport is unsuccessful, and less readily when it is “successful” (see text)	Must be capable of measuring the “success” (i.e. extent of coordination) but not the direction of the majority relative to one’s own behavioral state.
**“Informed”**	If in one of the active states, and if transport is successful, give up readily if going the opposite way as the majority and less readily if going the same way as the majority.	Must be capable of measuring (i) extent of coordination and (ii) preferred direction of the majority and must compare the latter to one’s own behavioral state.

Behavioral rules differ among different model runs, but within one run of the model all individuals are identical and have the same rules and parameter values. In “uninformed” groups, giving up rate constants, *G*_*L*_ and *G*_*R*_, are equal and do not change over the course of the transport effort. In “oblivious” and “informed” groups (defined in Table 1), realized giving up rates change over the course of the transport effort based on the abundances of individuals in the two active behavioral states (*N*_*R*_ and *N*_*L*_). Giving up depends on the “success” of transport. “Success” is operationally defined here as a high extent of coordination, measured as the absolute value of *N*_*R*_—*N*_*L*_ divided by the total number of individuals in the system. In other words, the extent of coordination is the degree to which individuals are *un*evenly distributed across the two active groups.

In oblivious groups individuals can measure success but they cannot detect if they are contributing to or detracting from that success. Individuals give up less frequently when | *N*_*L*_−*N*_*R*_ | is high, i.e. when there are many more individuals in one active state than the other, regardless of whether they are currently in the “right” or “left” state. Individuals are oblivious to their own contribution. If the transport is successful because many more individuals are trying to move the object to the left rather than the right, individuals moving right, who are going against the majority, still rarely give up. In ants, this would happen if they were capable of determining when the group sizes are uneven (or a proxy, such as the magnitude of the force on the object), but not in which direction. For example, this might occur if individuals are less likely to give up when they are moving, regardless of the direction.

In informed groups individuals are capable of detecting the same information as in the “oblivious” case, but additionally they can determine if their contribution is with or against the majority. Individuals give up less frequently when there is a higher extent of coordination only if their behavioral state matches the majority. For example, when *N*_*L*_−*N*_*R*_ is strongly positive, individuals in the “move left” state give up infrequently while individuals in the “move right” state give up quickly. As discussed above, *N*_*L*_−*N*_*R*_ is a measure of success that could be estimated by ants in multiple ways. For example, large values of *N*_*L*_−*N*_*R*_ (or highly negative values) will correspond to higher speeds over ground, which an ant might measure by estimating optical flow or her own leg movements.

**Fig 2:**
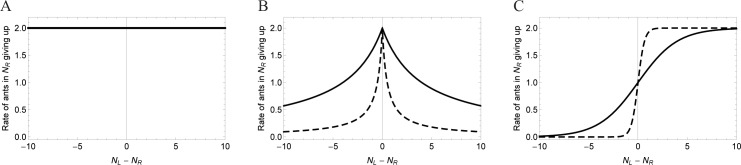
Giving-up rate constants for individuals in the “move right” behavioral state at various levels of success for each set of rules. The x-axis indicates a measure of success: the size difference between the two groups. (A) Uninformed rules, *a* = 2. (B) Oblivious rules, *g*_2_*/g*_1_ = 2, *g*_1_ = 4 (solid line) or 0.5 (dashed line). (C) Informed rules, *b*_1_ = 2, *b*_2_ = 0.5 (solid line) or 3 (dashed line). In (B) and (C), dashed lines indicate sharper shape parameters.

Equations governing the giving-up rate constants, *G*_*L*_ or *G*_*R*_, under each set of rules are listed in Table 2 and examples of how these functions behave are illustrated in Fig 2. In addition to the variables *N*_*R*_ and *N*_*L*_, functions for determining *G*_*L*_ and *G*_*R*_ depend on one or more parameters (Table 2). These parameters represent persistence and sensitivity, and are discussed below. We chose ranges of parameter values in order to manage computing time while selecting parameter ranges spanning multiple orders of magnitude. Some parameters were also constrained by necessity; for example, the shape parameter *g*_1_ must be non-zero.

**Table 2:** Functions governing the giving-up rate constants under each set of rules. Ranges of parameter values explored are in parentheses.

	*G*_*L*_	*G*_*R*_	Max *G* (Persistence^-1^)	Shape parameter
Uninformed (Fig 2A)	*a*	*a*	*a* (0.2–20)	*NA*
Oblivious (Fig 2B)	$\frac{g_{2}}{g_{1}+|N_{L}-N_{R}|}$	$\frac{g_{2}}{g_{1}+|N_{L}-N_{R}|}$	$\frac{g_{2}}{g_{1}}$ (0.2–20)	*g*_1_ (0.1–100)
Informed (Fig 2C)	$\frac{b_{1}}{1+e^{-b_{2}(N_{R}-N_{L})}}$	$\frac{b_{1}}{1+e^{-b_{2}(N_{L}-N_{R})}}$	*b*_1_ (0.2–20)	*b*_2_ (0.1–100)

#### Persistence and sensitivity

The giving up rates described above are tunable based on individuals’ persistence and sensitivity to information. These parameters govern the shape and maximum values of the giving-up functions (Fig 2). This maximum giving-up rate is the inverse of the engagement time under conditions when individuals give up fastest: when *N*_*L*_ = *N*_*R*_ in the oblivious case and when the difference between *N*_*L*_ and *N*_*R*_ is largest and opposed to the individual’s state in the informed case. We refer to this engagement time as persistence [20,40].

Persistence is individuals’ resistance to changing their behavior based on information [20], which could come from other individuals in the group, or other sources. Persistence can be measured in actual ants as the time it takes for an individual to give up or change the direction they are trying to move the object being carried. Highly persistent ants keep trying to move the object in the same direction for a long time, even without progress. On the other hand an ant with low persistence will try new strategies frequently, by pulling in different directions or even abandoning the effort temporarily or permanently. Intuitively, one expects a tradeoff for persistence. Groups with high persistence may have long-lasting deadlocks, while groups may also deadlock if no individual is persistent enough. We look at the effect of persistence in our model by varying the maximum possible giving-up rate (Fig 2). We ran the model with each of many maximum giving-up rate constants to examine the effect of persistence on extent of coordination; higher maximum giving-up rate constants mean lower persistence and vice versa (Table 2).

For the oblivious and informed cases we can also tune the parameters to change the sensitivity of individuals to the success of transport, that is, the magnitude of |*N*_*L*_−*N*_*R*_|. We do this by changing the shape of the giving-up functions through manipulations of the shape parameters (*g*_1_ and *b*_2_; Table 2), making the transition from low to high giving-up rate constants sharper or more gradual (Fig 2). With a gradual shape, when groups are relatively close to deadlocked (near *N*_*L*_ = *N*_*R*_), small changes in success lead to only small changes in the frequency of giving-up; individuals with a gradual shape therefore have low sensitivity to transport success. On the other hand, for sharp shapes, a small change in success when *N*_*L*_ ≈ *N*_*R*_ leads to a dramatic change in this frequency; this means individuals are highly sensitive. Differences in sensitivity could be caused by a number of factors, including error in sensing the group sizes. This shape parameter can be quantified for real organisms by fitting functions to data on individuals, for whom cooperative transport efficiencies are experimentally manipulated.

#### Differential equations

The model consists of the following set of differential equations giving the rates of change in the numbers of individuals in each behavioral state (moving left, moving right, or disengaged, respectively):

$$\frac{{dN}_{L}}{dt}=J_{L}N_{D}(t)-N_{L}(t)G_{L}$$ (1)

$$\frac{{dN}_{R}}{dt}=J_{R}N_{D}(t)-N_{R}(t)G_{R}$$ (2)

$$\frac{{dN}_{D}}{dt}=N_{L}(t)G_{L}+N_{R}(t)G_{R}-(J_{R}+J_{L})N_{D}(t)$$ (3)

where *N*_*D*_ is the number of individuals in the disengaged state (Fig 1). The ODEs are non-linear due to the dependence of *G*_*R*_ and *G*_*L*_ on *N*_*R*_ and *N*_*L*_. There is a constant number of total individuals (i.e., *N*_*D*_ *+ N*_*R*_ + *N*_*L*_ = *N* = constant), so this is a closed system, making the third differential equation implicit in the first two. Therefore in some cases we present results for the number of ants in the left and right states only. The ODE will always satisfy the following equations at equilibrium, where N* is the equilibrium abundance.

$$\frac{N_{L}^{*}}{N_{D}^{*}}=\frac{J_{L}}{G_{L}(N_{L}^{*},N_{R}^{*})}$$ (4)

$$\frac{N_{R}^{*}}{N_{D}^{*}}=\frac{J_{R}}{G_{R}(N_{L}^{*},N_{R}^{*})}$$ (5)

$$\frac{N_{L}^{*}}{N_{R}^{*}}=\frac{J_{L}}{J_{R}}\frac{G_{R}(N_{L}^{*},N_{R}^{*})}{G_{L}(N_{L}^{*},N_{R}^{*})}$$ (6)

In the uninformed and oblivious cases, *G*_*L*_ = *G*_*R*_, so Eq 6 simplifies to the following.

$$\frac{N_{L}^{*}}{N_{R}^{*}}=\frac{J_{L}}{J_{R}}$$ (7)

Because *G*_*R*_ and *G*_*L*_ are nonlinear functions of *N*_*R*_ and *N*_*L*_ in the oblivious and informed cases, it is difficult to solve this system of differential equations analytically. We numerically solved the ODE for each of nearly fifteen thousand sets of parameters, running the model under different sets of behavioral rules, global directional biases, and persistence and sensitivity. The range of parameter space explored for giving-up parameters is shown in Table 2. Additionally, we explored directional biases ranging from no bias, to joining rates of 0.01 and 0.9, respectively, for the two directions, a difference of two orders of magnitude; this range in bias provided a comprehensive illustration of the effect of joining bias. We then queried the results for particular metrics of interest, including the maximum extent of coordination on a direction (unevenness in the distribution of individuals across the left and right groups). We obtained numerical solutions using *Mathematica* (version 9.0.1.0) and we analyzed our results using *Mathematica* and R (RStudio version 0.98.977). All code is included in S1 Code. In addition to the numerical solutions, we analytically explored the stability of deadlocks in the informed case using fixed-point analysis [41] (see S1 Text).

### Stochastic Extension

Our ODE model makes certain assumptions required for any ODE, including instantaneous updating of information and continuous, rather than discrete, individuals. To test whether our conclusions are robust to these assumptions, and to look at the potentially important influence of stochasticity, we extended the model to a stochastic framework. The stochastic extension is an individual-based model operating in discrete time. We converted the instantaneous joining and giving-up rate constants (*J*_*L*_, *J*_*R*_, *G*_*L*_, and *G*_*R*_) to probabilities of joining or giving up in a given time step with the equation

$$P_{t}=1-e^{\left(-R\delta_{t}\right)}$$ (8)

where *P*_*t*_ is the probability of a behavioral shift in one time step, δ_t_ is the length of a time step (here, time steps were always unit length), and *R* is the instantaneous rate constant, either *J*_*L*_, *J*_*R*_, *G*_*L*_, or *G*_*R*_. We ran the stochastic simulation for 60 time steps; this duration was more than sufficient to capture transient dynamics. All other model assumptions and parameters were the same as in the deterministic model, including the three sets of rules.

In each time step we allow individuals to change their behavioral state. An active individual changes its status by giving-up with a probability equal to the giving-up probability for that individual’s current state (left or right), and disengaged individuals can change their status by joining. Because disengaged individuals can change their status in one of two ways (joining the left group or the right group), we first calculated the joint probability of an individual joining at all. For individuals that were to join, we then stochastically determined whether they joined left or right using the relative probabilities of each. We ran the stochastic model under the same parameter sets as the deterministic model, querying 1,000 simulations for each set of parameters. As with the deterministic model, we examined the extent of coordination. We performed and analyzed stochastic simulations in R (RStudio version 0.98.977). Code is included in S1 Code.

## Results

### Deterministic Model

**Fig 3:**
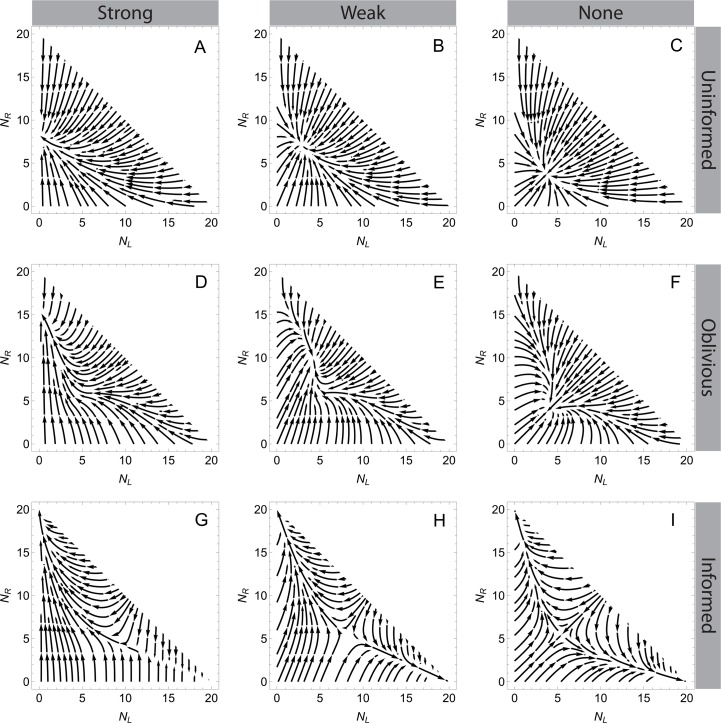
Streamplots of system dynamics. These show the direction the system tends towards for various abundances in each behavioral state. A-C: Uninformed rules, *a* = 1; D-F: Oblivious rules, *g*_1_ = 4, *g*_1_/*g*_2_ = 1; G-I: Informed rules, *b*_1_ = 1, *b*_2_ = 0.5. A, D, and G: Strong directional bias, *J*_*L*_ = 0.01, *J*_*R*_ = 0.7; B, E, and H: Weak directional bias, *J*_*L*_ = 0.3, *J*_*R*_ = 0.7; C, F, and I: No directional bias, *J*_*L*_ = *J*_*R*_ = 0.3.

Our primary measurement of success is the extent of coordination, which is the difference in the number of individuals in the active behavioral states (left and right) divided by the total number of individuals in the system. If the transport is uncoordinated, there are roughly equal numbers of individuals pulling each direction, and/or most individuals are disengaged. Streamplot representations of the vector fields portray the dynamical behavior of the system in Fig 3. Panels in the figure show different parameter sets, corresponding to each set of behavioral rules with differing directional biases. The streamplots indicate the direction the system tends towards starting from any possible combination of the numbers of individuals in each behavioral state (*N*_*L*_ and *N*_*R*_). The number of disengaged individuals, *N*_*D*_, is not shown explicitly because the total number of individuals is fixed at 20 (i.e., *N*_*D*_ = 20 –(*N*_*L*_ + *N*_*R*_)).

In the absence of a directional bias (*J*_*L*_ = *J*_*R*_), both the uninformed and oblivious rules have stable equilibria (Fig 3C and 3F). These are deadlocks, with equal numbers of individuals pulling left and right (as shown in Eq 7). Because they are stable, perturbations away from these equilibria lead back to them (Fig 3C and 3F). In other words, with no directional bias the uninformed and oblivious rules have deadlocks that cannot be broken. In informed groups, however, the equilibrium is unstable even if *J*_*L*_ = *J*_*R*_ (Fig 3I). If a deadlock occurs in this case, small perturbations grow exponentially, leading to convergence on one direction, which breaks symmetry. Although an unstable equilibrium occurs across most of the parameter space for informed groups, with small values of the shape parameter *b*_2_, the equilibrium is stable and deadlocks are maintained. Thus there is a critical value of *b*_2_ at which a phase transition occurs, from stable to unstable equilibrium. Using fixed-point analysis [41] we analytically determined that this critical value occurs when *b*_2_ has the following value:

$$b_{2}=\frac{b_{1}+4J}{2JN}$$ (9)

where J is the joining rate constant for each side (*J = J*_*L*_ = *J*_*R*_) and *N* is the total number of individuals in the system. This indicates that total group size affects deadlock breaking. Smaller groups require higher sensitivity (*b*_2_) to break deadlocks even in the informed case, and sensitivity is less important for large groups. Details of the fixed-point analysis are included in S1 Text.

When a directional bias is present (*J*_*L*_ ≠ *J*_*R*_) more individuals attempt to move the object in the direction favored by the bias, regardless of the set of rules (Fig 3, two left-most columns, also see Eqs 6 and 7). This is true regardless of the initial conditions for uninformed and oblivious groups; for informed groups, a large enough difference in the initial group sizes can overcome a joining bias (see rightmost portion of Fig 3G and 3H). The presence of a directional bias increases the extent of coordination, and, intuitively, strong biases lead to more coordination than weak ones (Fig 3 left column compared with middle column). However, for a given directional bias, individuals in informed groups are still more coordinated than individuals in uninformed or oblivious groups. Stable equilibria involving individuals working against one another still occur with a weak bias using these sets of rules (Fig 3B and 3E). With a sufficiently strong directional bias, in both uninformed and oblivious groups, the system moves to a state with almost no individuals going against the bias (Fig 3A and 3D), but there are still a substantial number of disengaged individuals who do not contribute to the effort (shown implicitly in Fig 3). This is because the disengaged group is constantly replenished by individuals giving up from the two active states. There are almost no disengaged individuals in informed groups. Thus, a directional bias allows for an unequal distribution of individuals between the two active states regardless of the behavioral rules, but the informed case still outperforms the other behavioral rules in that it maximizes engagement and the difference in group sizes.

### Stochastic Model

**Fig 4:**
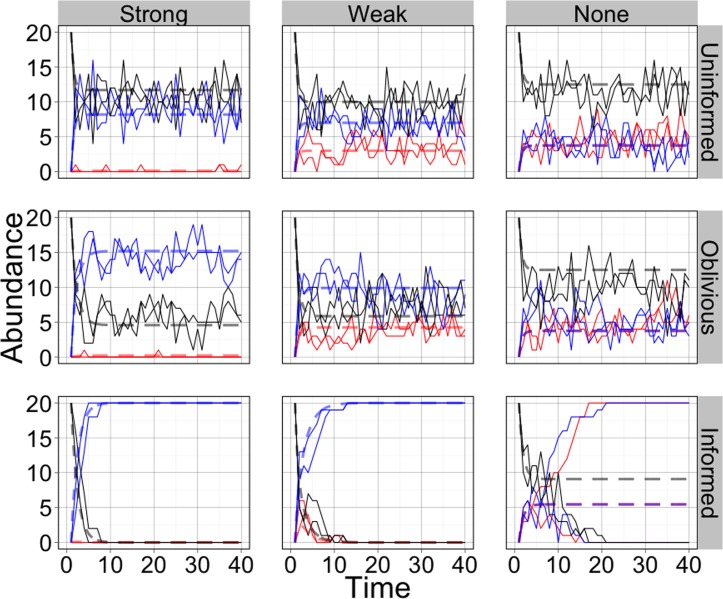
Abundance of ants in each behavioral state over time. Includes two example simulations with each set of parameters. Blue: number moving right, Red: number moving left, Black: number disengaged. Dashed lines show deterministic model behavior. Columns are different directional biases and rows are different sets of behavioral rules. The parameter values are the same as in the analogous panels in Fig 3. Uninformed rules: *a* = 1; oblivious rules: *g*_1_ = 4, *g*_1_/*g*_2_ = 1; informed rules: *b*_1_ = 1, *b*_2_ = 0.5. Strong directional bias: *J*_*L*_ = 0.01, *J*_*R*_ = 0.7; weak directional bias: *J*_*L*_ = 0.3, *J*_*R*_ = 0.7; no directional bias: *J*_*L*_ = *J*_*R*_ = 0.3.

**Fig 5:**
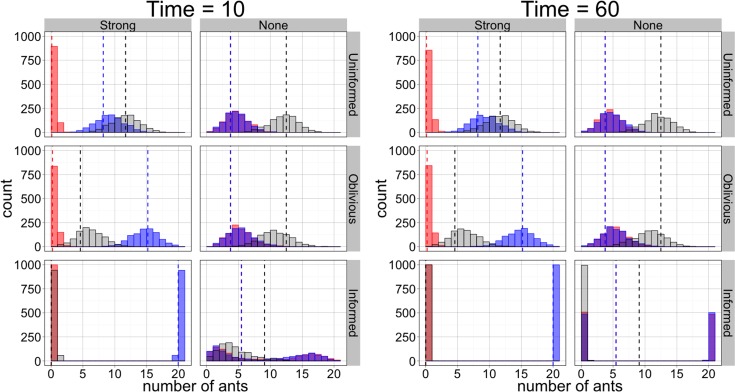
Histograms showing the state of 1,000 simulations at given time points. The x-axis shows the number of ants, and the y-axis shows the number of simulations for which the given behavioral state had that many ants at that time. Blue bars are for ants moving right, red bars are for ants moving left, and black bars are for disengaged ants. Bars appear purple when red and blue overlap. Dashed lines show the abundance of each behavioral state in the deterministic model. The parameter values are the same as in the analogous panels in Figs 3 and 4. Uninformed rules: *a* = 1; oblivious rules: *g*_1_ = 4, *g*_1_/*g*_2_ = 1; informed rules: *b*_1_ = 1, *b*_2_ = 0.5. Strong directional bias: *J*_*L*_ = 0.01, *J*_*R*_ = 0.7; no directional bias: *J*_*L*_ = *J*_*R*_ = 0.3.

The stochastic results are very similar to results from the deterministic model. Fig 4 shows the number of individuals in each behavioral state for two examples of stochastic simulations, under the same parameter sets shown in Fig 3. Fig 4 also includes the deterministic results. Deterministic and stochastic results match closely for each set of parameters except the informed case with no directional bias. This highlights the importance of stochasticity in this case. Without stochastic perturbations away from equilibrium groups remain deadlocked. In the stochastic model, perturbations are amplified, breaking symmetry and leading to consensus. Histograms of the behavior of the stochastic model across 1,000 simulations, at specific times, as well as deterministic results at those times, are shown in Fig 5 (also see S1 Movie).

In all other respects, deterministic and stochastic results were very similar despite differences in the formulations of these models. When a directional bias is present more individuals try to move the object in that direction than in the other direction under our initial conditions of all individuals beginning as disengaged. In the absence of a directional bias, roughly equal numbers of individuals are in each active state in uninformed and oblivious groups, while individuals converge on either direction in informed groups. In each of 1,000 simulations, the informed case allowed for convergence to a pure state (every individual or nearly every individual in the system transporting in the same direction) even with no directional bias (Fig 5 and S1 Movie). On the other hand, oblivious groups perform no better than uninformed groups, and neither of these sets of rules ever allowed for convergence on one direction.

When a directional bias is present, the informed case still leads to strikingly different performance than either of the other sets of rules. Individuals converge rapidly in informed groups, while in oblivious or uninformed groups, convergence, which we define as an increasing coordination through time until all individuals are pulling the same direction, does not occur. There are more individuals pulling in the direction of bias but coordination does not increase over time (Figs 4 and 5 and S1 Movie).

### Effect of Persistence and Sensitivity

**Fig 6:**
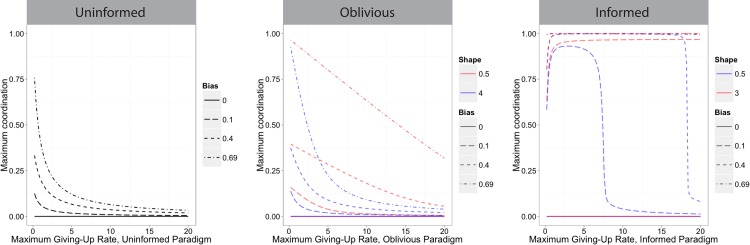
Effect of persistence (maximum engagement time, i.e. the inverse of the maximum giving-up rate constant) on maximum coordination. Maximum giving-up rate constant is the maximum possible as defined by the function (Table 2), actual values will depend on the number of individuals in each group. Extent of coordination is defined as the difference in the number of individuals pulling right and left, divided by the total number in the system. Maximum coordination is the maximum observed over a given time period; higher values on the y-axis indicate faster convergence. (A) uninformed rules, (B) oblivious rules, (C) informed rules. Lines with smaller dashes indicate larger directional bias, and the solid line indicates no bias (there is no coordination without a directional bias in the deterministic case). Red and blue lines indicate “sharp” and “gradual” shapes (sensitivities), respectively. Parameter values for shape match those in Fig 2.

Fig 6 shows the effect of persistence–or maximum engagement time–on the extent of coordination in the deterministic model for groups with total size fixed at 20 (see S1 Fig for results for other group sizes). The extent of coordination reported is the maximum observed over the time period evaluated. Parameter sets that converge more quickly on a direction will have a higher extent of coordination in that time period, and shorter deadlocks. Results in Fig 6 are therefore comparable across parameter sets, with higher agreement indicating more efficient transport. Because small perturbations away from equilibrium do not occur in the deterministic model, Fig 6 shows no coordination without directional bias.

The effect of persistence depends on the behavioral rules (Fig 6). In uninformed and oblivious groups, being highly persistent–having a low maximum giving-up rate constant–increases coordination (Fig 6A and 6B). In informed groups there is an optimal persistence value that maximizes coordination. The extent to which persistence affects coordination is stronger for small directional biases; at high directional biases there is a wide range of persistence values that result in high coordination. These results were not qualitatively different for different total group sizes, except that sensitivity, or sharpness of the giving-up function, was more and less important for smaller and larger groups, respectively (S1 Fig).

**Fig 7:**
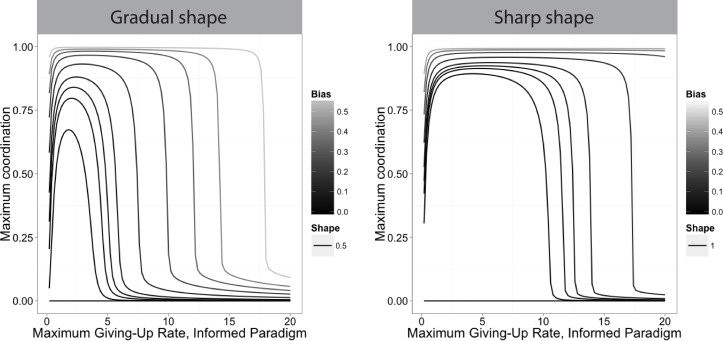
Effect of persistence (inverse of maximum giving-up rate constant) on maximum coordination in informed groups at low (gradual) shape values. Maximum giving-up rate constant is the maximum possible as defined by the function, actual values will depend on the number of individuals in each group. Extent of coordination is defined as the difference in the number of individuals pulling right and left, divided by the total number in the system. Maximum coordination is the maximum observed over a given time period, rather than an absolute maximum; higher values on the y-axis indicate faster convergence. Left column: shape parameter, *b*_2_ = 0.5, which corresponds to the solid line in Fig 2C. Right column: *b*_2_ = 1, which is less gradual.

For oblivious and informed groups, the sensitivity changes the effect of persistence (Figs 6C and 7); the uninformed case has no sensitivity parameter. In the oblivious case, sharper functions (lower values of *g*_1_) increase coordination for a given persistence value. In the informed case there is a critical sensitivity below which deadlocks cannot be broken, as discussed above and in S1 Text. This threshold depends on group size. Above this threshold, sharper functions (higher values of *b*_2_) further increase coordination, which has the effect of widening the range of persistence values that lead to coordination. For a moderate group size of 20 individuals, with a gradual shape and a small directional bias, there is a narrow range of persistence values that allow for high coordination. At small group sizes only groups with higher sensitivity or relatively strong directional bias coordinate successfully regardless of persistence, while large groups successfully coordinate across a wide range of persistence values regardless of sensitivity and bias (S1 Fig). Fig 7 shows in detail the extent of coordination for moderately sized groups with a wide range of directional biases and persistence values for two shape values, both relatively gradual (see S2 Fig for small and large groups).

## Discussion

Can relatively simple individuals with minimal information break deadlocks? Our results show that, indeed, individuals with simple behavioral rules and no memory can break deadlocks. However, only individuals in our informed case convincingly succeeded. These individuals followed simple rules: 1) give up more readily if one is moving against the majority and 2) do this to a greater extent for extreme majorities than slight majorities. Using these simple rules, with minimal information available, groups rapidly converge on a single travel direction, even when this required symmetry-breaking. Our deterministic and stochastic models agree, despite being formulated differently and having contrasting assumptions about individuals and time. This suggests that our conclusions are robust to specifics of model formulation.

In terms of information, it is sufficient for coordination for individuals to only be capable of measuring the direction that the majority of the group is trying to move the object and the relative sizes of the groups moving each direction (or a proxy). This information is crucial; with insufficient sensitivity to these group sizes (low *b*_2_) groups do not form a consensus. While sensitivity must be sufficient, it does not need to be high. As further discussed below, groups with only modest sensitivity were still coordinated across a wide range of other parameters. Thus, various proxies for relative group sizes may be accurate enough to break deadlocks. For example, individual ants could gain this information through stigmergy on the object being carried. If this is the case, a single sensory mode may provide all necessary information in informed groups. In nature, ants may have other information available, or may use different behavioral rules, but we show that by using these simple rules, groups are successful.

If individuals have global directional cues that correspond to a shared directional bias, this helps promote coordination regardless of the other information available. Additionally, if there is only one correct direction, for instance if there is a single nest entrance, a shared bias towards the nest would help ensure the group converges on the appropriate direction. But directional bias is neither necessary, nor sufficient, for convergence on a decision.

This makes sense considering the high variation in cooperative transport ability among ant species. We expect workers of all species to be good at knowing the direction of the nest. So we expect directional biases to be common among species, at least for situations with only one correct direction. Considering that efficient cooperative transport is comparatively rare among ants [25,30], the presence or absence of directional bias is not a good explanation for the observed variation in efficiency. On the other hand, the behavioral rule of giving up more readily when an individual is moving against the majority is a potential adaptation that dramatically improves efficiency. Future research should test whether efficient transporters have this adaptation.

We also investigated the effects that persistence and sensitivity (the sharpness of the giving-up function) have on coordination. These effects are complex and depend on the total group size and the behavioral rules. In the uninformed and oblivious cases, groups are most coordinated if individuals are highly persistent. While somewhat surprising, this makes sense in light of a tradeoff in persistence. Groups of highly persistent individuals may pull in opposing directions for a long time, but if movement does occur, either because of a directional bias or due to random fluctuations, the progress continues; they are unlikely to change their direction.

This suggests that high persistence allows species without other adaptations for cooperative transport, for instance those with behavioral rules similar to our uninformed or oblivious rules, to at least sometimes succeed at bringing a large object home to the nest. In such species, individuals are equally likely to give up whether they are helping or hurting the effort; even when successful movement occurs, individuals pulling with the motion may give up. High persistence makes it less likely that anyone will give up, allowing existing movement to continue. If, as in our model, individuals are identical, the individuals going the wrong way will also be unlikely to give up, so to minimize the length of deadlocks there should only be a small number of these individuals. A sufficient directional bias would accomplish this, and directional biases should be common in many circumstances (such as if the object is relatively far from the nest). So if high persistence is paired with a directional bias, it may allow ant species with rudimentary behavioral rules to conduct cooperative transport. Analogously, agents involved in any decision between two options, when they are unable to determine which option is winning, should be persistent to maximize the chance that a single option will be chosen.

In contrast to these results, in the informed case there is an optimum persistence value; groups with individuals more or less persistent than this value will be less coordinated. But the importance of persistence depends on directional bias, on the sharpness of the giving-up function, and on the total group size. In most of the parameter space of our model, the range of persistence values that lead to high coordination is wide. Only when the directional bias is low and the sensitivity is above the critical threshold but still gradual does one find a narrow peak in coordination around the optimum persistence. This was especially true for smaller group sizes. Large groups had a wide range of persistence values that would lead to coordination regardless of sensitivity, indicating that it may be easier to coordinate in a large group rather than a small group. This makes sense given that small groups will be more affected by the behavior of single individuals. In order for informed individuals in groups of small to moderate size to be highly coordinated, they must have one, but do not need more than one, of the following: high directional bias, high sensitivity to the sizes of the two groups, or finely-tuned persistence. Each of these is a potential adaptation for efficient cooperative transport in informed groups. This flexibility makes the behavioral rules in the informed case relatively robust to deficiencies in the individuals’ capabilities as long as they have at least minimal accuracy in sensing group sizes.

Because we did not constrain our model by tuning it to a particular species, our results are applicable to other collective decisions. A system in which groups must decide among multiple options is vulnerable to deadlocks, especially when the options are relatively equal (analogous to having no directional bias); small group size may also make deadlocks more likely. One of the best studied examples of collective decisions is nest-site selection in social insects (reviwed in [6]). As discussed above, some recent work on the “stop-signal” in honeybees focuses on how this signal prevents deadlocks in nest-site selection [9,14,15].

The outcome of our deterministic model with respect to the effect of behavioral rules looks similar to the results of Seeley et al. [9] and Pais et al. [14], who each investigated decision-making dynamics in honeybee nest site selection with similar models. For example, compare Fig 3 here to Fig 3C in [9] and the inset in Fig 2 in [14]. Both models investigate the accumulation of “votes” for one of two, mutually exclusive choices in a decision, and in each case the number of individuals aligned with the two options determines which option is chosen. A key difference between the models, however, involves the timing of the decision. In honeybee nest-site selection, a decision is reached when a quorum of scouts is present at one of the potential nests [7]. In cooperative transport, an initial decision is reached when the difference between the number of individuals in each group reaches a certain threshold–enough to begin movement–rather than when the absolute number of individuals in a particular group is high. Perhaps a more important difference between these models relates to communication. Unlike our model the Seeley et al. [9] and Pais et al. [14] models include direct communication among individuals. Honeybee scouts actively advertise for a particular nest site (a positive feedback mechanism) and stop other scouts from advertising for a different site using the stop signal (a negative feedback mechanism) [35]. Our model produces similar dynamics using a simpler mechanism. In informed groups, positive and negative feedbacks are combined into a single mechanism that requires no signals. An individual is less likely to give up if her faction is large compared to the other faction (positive feedback), and more likely to give up if the opposite is true (negative feedback, analogous to cross-inhibition). Informed individuals only need to measure the relative group sizes to make effective decisions.

The Seeley et al. [9] and Pais et al. [14] models elegantly and realistically reproduce the dynamics of nest-site selection in honeybees. Our model is simpler, yet produces similar dynamics in terms of the accumulation of votes for a single option, indicating that direct communication among individuals is not necessary for a decision in the case of cooperative transport. The fact that some of our results are similar lends credence to the idea that results from one collective decision-making system can be generalizable to others. Among collective systems, social insects are uncommonly apt for experiment, since individuals are easily observed and manipulated. Because lessons are transferable across at least some systems, we can use social insects as model systems for other systems that are harder to study, such as neuronal networks.

Our model demonstrates that simple behavioral rules can lead to a consensus about travel direction during cooperative transport, even without a directional bias. Our simulated ants had no memory, limited sensory ability, and followed only simple rules, yet made decisions rapidly in informed groups. We identify a potential adaptation–giving up more readily when going against the majority–that allows for deadlock-breaking, and may explain why we see such large variation in cooperative transport ability among ant species. While it is currently not possible to directly measure this adaptation in ants, the consequences we have modeled here can, and should, be measured to see if real ants use this behavioral rule. Our model reproduces dynamics similar to those of other decision-making processes [9,14], and our conclusions are generalizable to other collective decisions. Though cooperative transport is a challenging task that requires coordination, behavioral complexity is not a prerequisite for success.

## Supporting Information

S1 Code Coding supplement (zip file). All code is included here. S1 Code also includes example parameter sets necessary to reproduce Figs 3 through 7.
(ZIP)

S1 Fig Persistence effects at different group sizes. Effect of persistence (inverse of maximum giving-up rate constant) on maximum coordination for small, moderate, and large groups. Maximum giving-up rate constant is the maximum possible as defined by the function, actual values will depend on the number of individuals in each group. Extent of coordination is defined as the difference in the number of individuals pulling right and left, divided by the total number in the system. Maximum coordination is the maximum observed over a given time period, rather than an absolute maximum; higher values on the y-axis indicate faster convergence. Top row: uninformed rules, middle row: oblivious rules, bottom row: informed rules. Left column: total group size = 6, middle column: total group size = 20, right column: total group size = 200. Lines with smaller dashes indicate lower directional bias.
(TIF)

S2 Fig Persistence and sensitivity effects in informed case at different group sizes. Effect of persistence (inverse of maximum giving-up rate constant) on maximum coordination in small, moderate, and large informed groups at low (gradual) shape values. Maximum giving-up rate constant is the maximum possible as defined by the function, actual values will depend on the number of individuals in each group. Extent of coordination is defined as the difference in the number of individuals pulling right and left, divided by the total number in the system. Maximum coordination is the maximum observed over a given time period, rather than an absolute maximum; higher values on the y-axis indicate faster convergence. Top row: shape parameter, *b*_2_ = 0.5, which corresponds to the solid line in fic. 2C. Bottom row: *b*_2_ = 1, which is less gradual. Left column: total group size = 6, middle column: total group size = 20, right column: total group size = 200.
(TIF)

S1 Movie Simulation results. Movie of histograms showing the state of 1,000 simulations of the stochastic model for each of nine parameter sets. Each frame is a time point. Each panel is a set of parameter values, corresponding to the analogous panels in Figs 3 and 4. Blue bars show the number of simulations for which the number of ants moving right had that abundance. Red bars are for ants moving left, and black bars are for disengaged ants. Bars appear purple when red and blue overlap.
(MP4)

S1 Text Analysis of deadlock stability in informed case. (DOCX)

## References

[1] Camazine S, Deneubourg J-L, Franks NR, Sneyd J, Theraulaz G, Bonabeau E. Self-Organization in Biological Systems. Princeton University Press; 2001. 538 p.

[2] Conradt L, Roper TJ. Consensus decision making in animals. Trends Ecol Evol. 2005 8;20(8):449–56. 16701416 10.1016/j.tree.2005.05.008

[3] Couzin ID, Krause J, Franks NR, Levin SA. Effective leadership and decision-making in animal groups on the move. Nature. 2005 2 3;433(7025):513–6. 15690039 10.1038/nature03236

[4] Sumpter DJT, Zabzina N, Nicolis SC. Six Predictions about the Decision Making of Animal and Human Groups. Manag Decis Econ. 2012 7 1;33(5–6):295–309.

[5] Zabzina N, Dussutour A, Mann RP, Sumpter DJT, Nicolis SC. Symmetry Restoring Bifurcation in Collective Decision-Making. PLoS Comput Biol. 2014 12 18;10(12). 10.1371/journal.pcbi.1003960 PMC4270427 25521109

[6] Visscher PK. Group Decision Making in Nest-Site Selection Among Social Insects. Annu Rev Entomol. 2007;52(1):255–75. 16968203 10.1146/annurev.ento.51.110104.151025

[7] Seeley TD. Honeybee democracy Princeton, New Jersey: Princeton University Press; 2010.

[8] Sasaki T, Pratt SC. Emergence of group rationality from irrational individuals. Behav Ecol. 2011 4;22(2):276–81.

[9] Seeley TD, Visscher PK, Schlegel T, Hogan PM, Franks NR, Marshall JAR. Stop Signals Provide Cross Inhibition in Collective Decision-Making by Honeybee Swarms. Science. 2012 1 6;335(6064):108–11. doi: 10.1126/science.1210361 22157081 10.1126/science.1210361

[10] Marshall JAR, Bogacz R, Dornhaus A, Planqué R, Kovacs T, Franks NR. On optimal decision-making in brains and social insect colonies. J R Soc Interface. 2009 1 1. 10.1098/rsif.2008.0511 PMC2827444 19324679

[11] Reid CR, Garnier S, Beekman M, Latty T. Information integration and multiattribute decision making in non-neuronal organisms. Anim Behav. 2015 2;100:44–50.

[12] Czaczkes TJ, Grüter C, Ratnieks FLW. Trail Pheromones: An Integrative View of Their Role in Social Insect Colony Organization. Annu Rev Entomol. 2015;60(1):581–99. 25386724 10.1146/annurev-ento-010814-020627

[13] Lindauer M. Schwarmbienen auf Wohnungssuche. Z Vgl Physiol. 1955;37:263–324.

[14] Pais D, Hogan PM, Schlegel T, Franks NR, Leonard NE, Marshall JAR. A Mechanism for Value-Sensitive Decision-Making. Plos One. 2013 9 2;8(9). 10.1371/journal.pone.0073216 PMC3759446 24023835

[15] Niven JE. How Honeybees Break a Decision-Making Deadlock. Science. 2012 1 6;335(6064):43–4. doi: 10.1126/science.1216563 22157084 10.1126/science.1216563

[16] Deneubourg JL, Goss S, Franks N, Pasteels JM. The blind leading the blind: Modeling chemically mediated army ant raid patterns. J Insect Behav. 2(5):719–25.

[17] Vries H de Biesmeijer JC. Self-organization in collective honeybee foraging: emergence of symmetry breaking, cross inhibition and equal harvest-rate distribution. Behav Ecol Sociobiol. 2002 3 27;51(6):557–69.

[18] Hamann H, Schmickl T, Wörn H, Crailsheim K. Analysis of emergent symmetry breaking in collective decision making. Neural Comput Appl. 2010 4 28;21(2):207–18.

[19] Sumpter DJT. Collective Animal Behavior. Princeton University Press; 2010. 302 p.

[20] McCreery HF, Breed MD. Cooperative transport in ants: a review of proximate mechanisms. Insectes Sociaux. 2014 5;61(2):99–110.

[21] Hölldobler B, Stanton RC, Markl H. Recruitment and food-retrieving behavior in Novomessor (Formicidae, Hymenoptera). Behav Ecol Sociobiol. 1978;4(2):163–81.

[22] Wojtusiak J, Godzinska E, Dejean A. Capture and retrieval of very large prey by workers of the African weaver ant, Oecophylla longinoda (Latreille 1802). Trop Zool. 1995 11;8(2):309–18.

[23] Franks NR, Sendova-Franks AB, Simmons J, Mogie M. Convergent evolution, superefficient teams and tempo in Old and New World army ants. Proc R Soc B Biol Sci. 1999 8 22;266(1429):1697–1697.

[24] Berman S, Lindsey Q, Sakar MS, Kumar V, Pratt SC. Experimental Study and Modeling of Group Retrieval in Ants as an Approach to Collective Transport in Swarm Robotic Systems. Proc IEEE. 2011 9;99(9):1470–81.

[25] Czaczkes TJ, Ratnieks FLW. Cooperative transport in ants (Hymenoptera: Formicidae) and elsewhere. Myrmecol News. 2013;18:1–11.

[26] Moffett MW. Adventures among Ants: A Global Safari with a Cast of Trillions. 1st ed. University of California Press; 2010. 288 p.

[27] Kumar GP, Buffin A, Pavlic TP, Pratt SC, Berman SM. A Stochastic Hybrid System Model of Collective Transport in the Desert Ant Aphaenogaster Cockerelli. In: Proceedings of the 16th International Conference on Hybrid Systems: Computation and Control. New York, NY, USA: ACM; 2013. p. 119–24. (HSCC ‘13).

[28] Wilson S, Pavlic TP, Kumar GP, Buffin A, Pratt SC, Berman S. Design of ant-inspired stochastic control policies for collective transport by robotic swarms. Swarm Intell. 2014 10 31;8(4):303–27.

[29] Gelblum A, Pinkoviezky I, Fonio E, Ghosh A, Gov N, Feinerman O. Ant groups optimally amplify the effect of transiently informed individuals. Nat Commun. 2015 7 28;6. 10.1038/ncomms8729 PMC4525283 26218613

[30] Moffett M. Ant Foraging. Res Explor. 1992 SPR;8(2):220–31.

[31] Servedio MR, Brandvain Y, Dhole S, Fitzpatrick CL, Goldberg EE, Stern CA, et al Not Just a Theory—The Utility of Mathematical Models in Evolutionary Biology. PLoS Biol. 2014 12 9;12(12). 10.1371/journal.pbio.1002017 PMC4260780 25489940

[32] Couzin ID, Krause J, James R, Ruxton GD, Franks NR. Collective Memory and Spatial Sorting in Animal Groups. J Theor Biol. 2002 9 7;218(1):1–11. 12297066 10.1006/jtbi.2002.3065

[33] Lerman K, Martinoli A, Galstyan A. A Review of Probabilistic Macroscopic Models for Swarm Robotic Systems In: Şahin E, Spears WM, editors. Swarm Robotics. Springer Berlin Heidelberg; 2005 p. 143–52. (Lecture Notes in Computer Science).

[34] Correll N, Martinoli A. Modeling and designing self-organized aggregation in a swarm of miniature robots. Int J Robot Res. 2011 4 1;30(5):615–26.

[35] Dornhaus A. Finding optimal collective strategies using individual-based simulations: colony organization in social insects. Math Comput Model Dyn Syst. 2012;18(1):25–37.

[36] Åkesson S, Wehner R. Visual navigation in desert ants Cataglyphis fortis: are snapshots coupled to a celestial system of reference? J Exp Biol. 2002 7 15;205(14):1971–8. 12089203 10.1242/jeb.205.14.1971

[37] Hölldobler B, Wilson EO. The Ants. 1st ed. Belknap Press of Harvard University Press; 1990. 732 p.

[38] Pielstroem S, Roces F. Vibrational communication in the spatial organization of collective digging in the leaf-cutting ant Atta vollenweideri. Anim Behav. 2012 10;84(4):743–52.

[39] Kube C, Bonabeau E. Cooperative transport by ants and robots. Robot Auton Syst. 2000 1 31;30(1–2):85–101.

[40] Czaczkes TJ, Nouvellet P, Ratnieks FLW. Cooperative food transport in the Neotropical ant, Pheidole oxyops. Insectes Sociaux. 2011;58(2):153–61.

[41] Strogatz SH. Nonlinear dynamics and chaos Perseus Books Publishing; 1994. 498 p. (Studies in Nonlinearity).

