# Supplementary material for: Consensus or Deadlock? Consequences of Simple Behavioral Rules for Coordination in Group Decisions
Source: PLoS One. 2016 Sep 28;11(9):e0162768. doi: 10.1371/journal.pone.0162768 (PMC5040253; doi:10.1371/journal.pone.0162768)
Supplement: S1 Code — All code is included here. S1 Code also includes example parameter sets necessary to reproduce Figs 3 through 7. (ZIP) [file pone.0162768.s001.zip › S1code/0-readMe.rtf]

Cooperative Transport Model In general, if you open coding files from the “coding supplement” folder, it will just run. If you move them to other places you may need to change working directories. In the coding supplement zip file, you should find this file, as well the following as 5 codes:	1: the mean field model guts (Mathematica notebook)	2: the stochastic model guts (R file)	3: the code to create figure 3 (Mathematica notebook)	4: the code to create figures 4, 5, and movie S1 (R file)	5: the code to create figures 6 and 7 (R file)Additionally there should be the following folders:	- movieFrames: a blank folder. If you want to create a movie like S2 movie, each frame will be saved in this folder.	- outputFiles: data from the models will be saved as csv files in here	- parameterFiles: this folder contains csv files with the parameter spaces for the figures in the manuscript. Use the same format to make your own parameter spaces.  Workflow1. Make a parameter space you want to look at (or use one of the existing ones). When you have done this, you can go ahead and run files 1, 2, or 3. All of these files require only a parameter space as external information.2. If you ran files 1 or 2, there should now be data files in the outputFiles folder. You can now run files 4 and 5. These files create figures similar to those in the manuscript. 3. File 4 should be run with stochastic data from File 2 (set up best using data made from parameter file “paramsForFigs345”). File 5 should be run with deterministic data from File 1 (set up best using two sets of data from parameter files “paramsForFig6” and “paramsForFig7”).
