# Supplementary material for: Consensus or Deadlock? Consequences of Simple Behavioral Rules for Coordination in Group Decisions
Source: PLoS One. 2016 Sep 28;11(9):e0162768. doi: 10.1371/journal.pone.0162768 (PMC5040253; doi:10.1371/journal.pone.0162768)
Supplement: S1 Code — All code is included here. S1 Code also includes example parameter sets necessary to reproduce Figs 3 through 7. (ZIP) [file pone.0162768.s001.zip › S1code/parameterFiles/0-readMePars.rtf]

Parameter setsThis folder contains three files of parameter sets, which were used to create the figures in the manuscript. During model exploration, a much larger range of parameters was tested. Use these parameter files to explore the model, or make your own following the same structure. 
