# Supplementary figures and images for: Consensus or Deadlock? Consequences of Simple Behavioral Rules for Coordination in Group Decisions
Source: PLoS One. 2016 Sep 28;11(9):e0162768. doi: 10.1371/journal.pone.0162768 (PMC5040253; doi:10.1371/journal.pone.0162768)

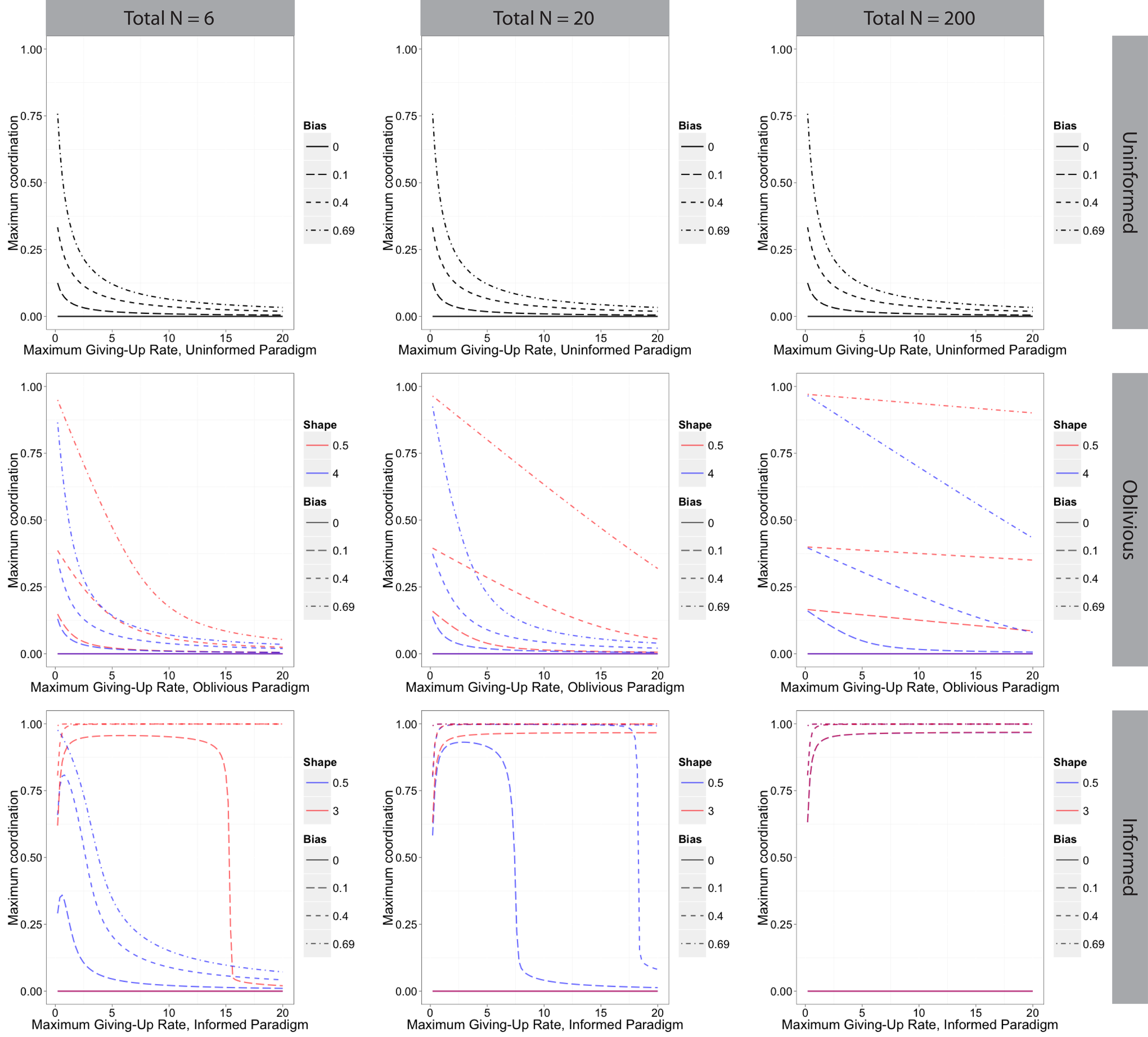

Supplement: S1 Fig — Effect of persistence (inverse of maximum giving-up rate constant) on maximum coordination for small, moderate, and large groups. Maximum giving-up rate constant is the maximum possible as defined by the function, actual values will depend on the number of individuals in each group. Extent of coordination is defined as the difference in the number of individuals pulling right and left, divided by the total number in the system. Maximum coordination is the maximum observed over a given time period, rather than an absolute maximum; higher values on the y-axis indicate faster convergence. Top row: uninformed rules, middle row: oblivious rules, bottom row: informed rules. Left column: total group size = 6, middle column: total group size = 20, right column: total group size = 200. Lines with smaller dashes indicate lower directional bias. (TIF) [file pone.0162768.s002.tif]

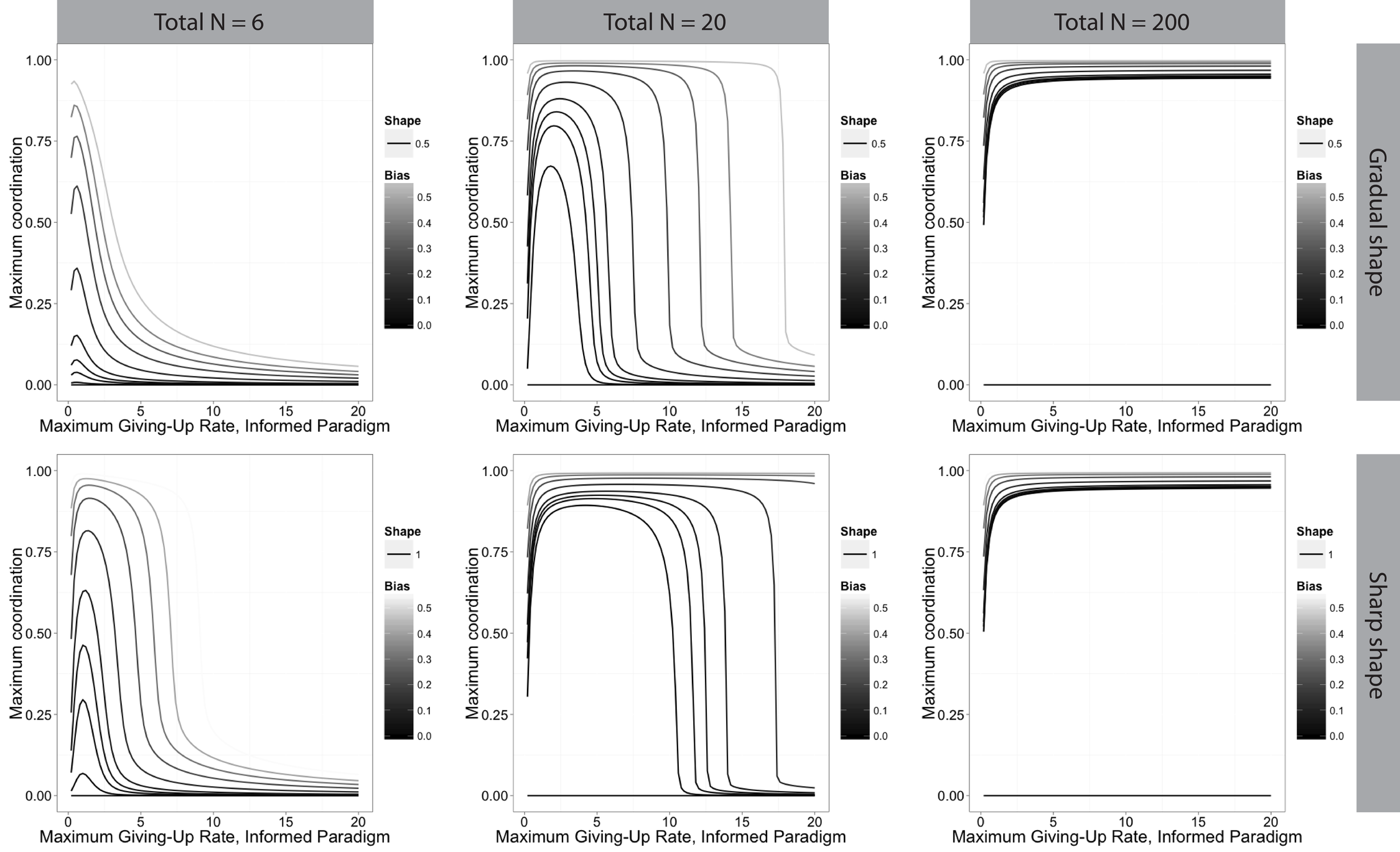

Supplement: S2 Fig — Effect of persistence (inverse of maximum giving-up rate constant) on maximum coordination in small, moderate, and large informed groups at low (gradual) shape values. Maximum giving-up rate constant is the maximum possible as defined by the function, actual values will depend on the number of individuals in each group. Extent of coordination is defined as the difference in the number of individuals pulling right and left, divided by the total number in the system. Maximum coordination is the maximum observed over a given time period, rather than an absolute maximum; higher values on the y-axis indicate faster convergence. Top row: shape parameter, b2 = 0.5, which corresponds to the solid line in fic. 2C. Bottom row: b2 = 1, which is less gradual. Left column: total group size = 6, middle column: total group size = 20, right column: total group size = 200. (TIF) [file pone.0162768.s003.tif]
