# Supplementary material for: Consensus or Deadlock? Consequences of Simple Behavioral Rules for Coordination in Group Decisions
Source: PLoS One. 2016 Sep 28;11(9):e0162768. doi: 10.1371/journal.pone.0162768 (PMC5040253; doi:10.1371/journal.pone.0162768)
Supplement: S1 Text — (DOCX) [file pone.0162768.s005.docx]

**S1 Text**

**Analysis of deadlock stability in informed case**

We used fixed-point analysis to analytically investigate the stability of deadlocks in the informed case ([34] Section 6.3, also described elsewhere). First, we found the location of the fixed point (equilibrium) in terms of *N_L_* and *N_R_*. The fixed-point is the location where the time derivatives of *N_L_* and *N_R_* are equal to zero. We found that the fixed point occurs at the following values of *N_L_* and *N_R_*:

$$N_{L}^{*}=\frac{2 J_{L} N_{total}}{b_{1}+4 J_{L}} , N_{R}^{*}=\frac{2 J_{R} N_{total}}{b_{1}+4 J_{R}}$$

where *N_L_** and *N_R_** are the specific values at the fixed point. *J_L_* and *J_R_* are the transition rates for individuals moving from the disengaged group to the “moving left” and “moving right” groups, respectively. We made the simplification that *N_L_* = *N_R_* to examine only the case of a deadlock. Note that this condition of *N_L_** *= N_R_** is only satisfied if *J_L_ = J_R_*. Thus, deadlocks with equal numbers in each group only occur with no directional bias.

Next, we performed a first order Taylor expansion around the fixed point, with $\vec{\Delta}=\left( \begin{matrix} \Delta_{1} \\ \Delta_{2} \end{matrix} \right)$ as a perturbation, where $\left( \begin{matrix} 1 \\ 0 \end{matrix} \right)$ and $\left( \begin{matrix} 0 \\ 1 \end{matrix} \right)$ are the *N_L_* and *N_R_* directions, respectively. The equations of motion for $\vec{\Delta}$ are:

$$\frac{d\Delta_{1}(t)}{dt}=-\frac{\left( b_{1}^{2}+8 J^{2}+b_{1} J\left( 6-b_{2} N_{total} \right) \right)\Delta_{1}\left( t \right)+J \left( 8 J+b_{1}\left( 2+b_{2} N_{total} \right) \right)\Delta_{2}(t)}{2 (b_{1}+4 J)}$$

$$\frac{d\Delta_{2}(t)}{dt}=-\frac{J \left( 8 J+b_{1}\left( 2+b_{2} N_{total} \right) \right)\Delta_{1}\left( t \right)+\left( b_{1}^{2}+8 J^{2}+b_{1} J\left( 6-b_{2} N_{total} \right) \right)\Delta_{2}\left( t \right)}{2 (b_{1}+4 J)}$$

This set of coupled differential equations is of the form:

$$\frac{d\vec{\Delta}}{dt}=M\vec{\Delta} \mathrm{where} M=\left( \begin{matrix} a & b \\ b & a \end{matrix} \right)$$

$$a=-\frac{\left( b_{1}^{2}+8 J^{2}+b_{1} J\left( 6-b_{2} N_{total} \right) \right)}{2 \left( b_{1}+4 J \right)} \mathrm{and} b=-\frac{J \left( 8 J+b_{1}\left( 2+b_{2} N_{total} \right) \right)}{2 \left( b_{1}+4 J \right)} .$$

The eigenvectors of this system are $\left( \begin{matrix} 1 \\ 1 \end{matrix} \right)$, when Δ_1_ = Δ_2_ and $\left( \begin{matrix} 1 \\ -1 \end{matrix} \right)$, when Δ_1_ = -Δ_2_. The corresponding eigenvalues are λ_1_ = (*a* + *b*) and λ_2_ = (*a – b*), which are equal to:

$$\lambda_{1}=-\frac{b_{1}}{2} - 2 J \lambda_{2}=b_{1}\left( -\frac{1}{2}+\frac{b_{2} J N_{total}}{b_{1}+4 J} \right)$$

The eigenvalue λ_1_ is always negative, so perturbations along the $\left( \begin{matrix} 1 \\ 1 \end{matrix} \right)$ eigenvector decay exponentially back to the fixed point for all parameter values. On the other hand, λ_2_ can be negative or positive. When this eigenvalue is positive, perturbations along the $\left( \begin{matrix} 1 \\ -1 \end{matrix} \right)$ eigenvector grow exponentially, breaking symmetry. The λ_2_ eigenvector switches from negative to positive at the following critical value of *b*_2_:

$$b_{2}=\frac{b_{1}+4J}{2JN_{total}} .$$

When *b*_2_ is smaller than this value, the fixed point is a stable equilibrium, and the system maintains deadlocks. When *b*_2_ is larger than this value, the fixed point is an unstable saddle point; small perturbations grow exponentially, allowing for deadlock-breaking.
